# Supplementary material for: Transcriptomic Insights into Mechanisms of Early Seed Maturation in the Garden Pea (Pisum sativum L.)
Source: Cells. 2020 Mar 23;9(3):779. doi: 10.3390/cells9030779 (PMC7140803; doi:10.3390/cells9030779)

Zhewan−1(25) vs Zhon

Global Summary

%DE = 0.87

genes with fdr < 0.2 = 553 (230 + / 323 −)

genes with fdr < 0.1 = 501 (206 + / 295 −)

genes with fdr < 0.05 = 447 (176 + / 271 −)

genes with fdr < 0.01 = 373 (147 + / 226 −)

genes in genesets = 0

<FC> = 0

<t−score> = −25670.75

<p−value> = 0

<fdr> = 0.14

Global Genelist

Rank	ID	log(FC)	p−value	fdr	Metagene	Description
1	TRINITY_DN1	−15.24	0e+00	0.00000020 x 1		
2	TRINITY_DN6	1.18	0e+00	0.00000020 x 20		
3	TRINITY_DN3	18.79	2e−09	0.0000009 x 20		
4	TRINITY_DN1	0.52	2e−08	0.0000009 x 20		
5	TRINITY_DN4	0.16	2e−08	0.0000009 9 x 16		
6	TRINITY_DN5	0.6	3e−08	0.0000023 x 20		
7	TRINITY_DN1	2.11	6e−08	0.0000021 x 20		
8	TRINITY_DN4	0.49	1e−07	0.0000021 4 x 20		
9	TRINITY_DN3	−0.05	1e−07	0.0000049 5 x 10		
10	TRINITY_DN1	−0.17	2e−07	0.0000049 8 x 10		
11	TRINITY_DN6	0	2e−07	0.0000062 0 x 10		
12	TRINITY_DN6	0	3e−07	0.0000079 x 11		
13	TRINITY_DN5	0.3	5e−07	0.0000078 x 11		
14	TRINITY_DN2	0.17	7e−07	0.0000079 x 18		
15	TRINITY_DN4	0	1e−06	0.0000079 x 11		
16	TRINITY_DN1	5.39	1e−06	0.0000078 x 1		
17	TRINITY_DN6	0	1e−06	0.0000079 x 11		
18	TRINITY_DN3	0.22	1e−06	0.0000078 x 15		
19	TRINITY_DN3	0.34	1e−06	0.0000078 x 1		
20	TRINITY_DN4	−1.36	1e−06	0.0000078 5 x 3		

Portrait

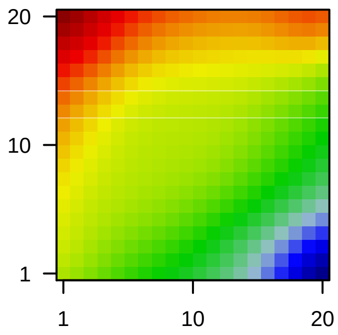

Top 100 DE genes

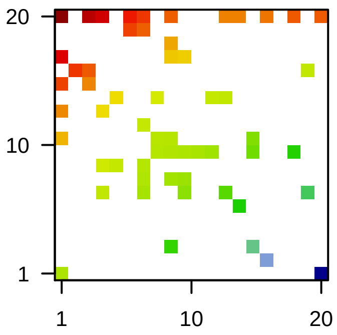

p−values

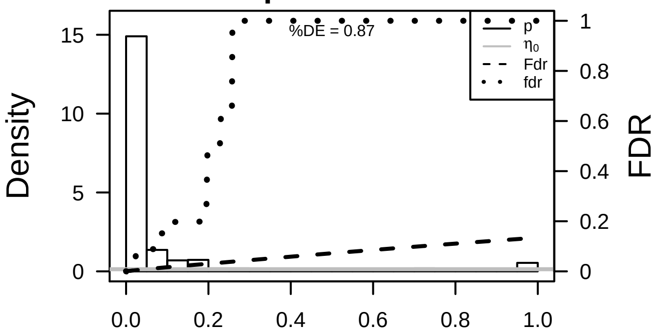

Supplement: Supplementary file 1 [file cells-09-00779-s001.zip › Supplementary materials/FileS3.zip!/FileS2/Hormones - Results/Summary Sheets - Differences/Reports/Zhewan.1.25..vs.Zhongwan.6.10..pdf]
